# Nurses' Accommodation at Glasgow Hospitals

**Published:** 1915-11-27

**Authors:** 


### NURSES' ACCOMMODATION AT GLASGOW HOSPITALS.

### An Educative Contrast and a Revelation.

In The Hospital of November 14 and 28, 1914, we gave a very full report of the new Royal Infirmary buildings at Glasgow, which were opened by the King and Queen on July 7. This was the second occasion on which we had been present at visits paid by their Majesties to Glasgow hospitals, and we noted that both the King and Queen took a great personal interest in inspecting the portions of the hospitals which they were shown. We hope in due course Her Majesty Queen Mary may visit Glasgow again, and that she may make it her business to inspect personally the accommodation provided for the nurses at the Royal Infirmary, the Royal Hospital for Sick Children, and the Western Infirmary, Glasgow. We make this suggestion because a thorough inspection which we have made of the nurses' accommodation provided at the three Glasgow hospitals just mentioned exhibited such contrasts and striking differences as to be well-nigh incomprehensible.

### Want of Artistic Taste.

The only thing we could find in the Royal Hospital for Sick Children, for instance, that really is unworthy of our newest, latest, and most interesting children's hospital is the lamentable evidence of the want of artistic taste and practical knowledge exhibited by the furnishing and equipment of the nurses' home. The same remark applies with equal force to parts of the nurses' home of the Royal Infirmary; but this latter is an older building, and we look forward to the advent of some wealthy Glasgow man who will visit Mr. Hedderwick, the chairman, and offer him a cheque for £5,000 or £10,000 to be devoted entirely to the equipment of the new infirmary with the newest and latest appliances and the most artistic fittings and furniture for its nurses' home. We devoted many hours to these two institutions, and it is only true to say that the lost opportunities for creating an inspiriting and health-giving atmosphere through the rooms provided for the relaxation and enjoyment of the nurses during their hours off duty depressed us more than anything we can call to mind. The will to do good deeds by giving this atmosphere was, we are sure, present to the minds of those who undertook the duty of selecting the furniture and fittings, but no persons should be entrusted with such a duty unless they have the gifts to discharge it with the maximum of efficiency.

### A Contrast.

Almost broken-hearted, we decided to go to the Western Infirmary without notice, and see exactly whether or not there was a hospital in Glasgow where the nursing staff had the immense asset of a bright atmosphere and an artistic tone to cheer and inspirit them, however unconsciously, after the hours of work were over. The result was magnetic upon ourselves, and we commend to anyone interested a visit to the nursing homes of these three institutions that they may appreciate at its true value the relative excellence or otherwise of these sections of the Glasgow hospitals.

Her Majesty Queen Mary, like the King, is always learning and always anxious to take every opportunity to acquire useful information which tends to help the formation of an accurate judgment of the actual condition of affairs and of the administration and conduct of a great hospital or other public institution. So their Majesties' inspections, as they increase and multiply, possess for our King and Queen never-failing sources of fresh interest, whilst they contribute increased knowledge of great practical value to the best interests of the people of these realms. Hospital managers throughout Glasgow are keenly competitive in the battle for efficiency of administration.

Much as we desire to secure the sweeping away and change in some of the nurses' accommodation which we have indicated, so that a bright atmosphere and artistic and cheerful surroundings may be equally present in each of the three great hospitals of Glasgow, it will be a disappointment should it not be possible for Queen Mary to take an early opportunity to inspect the nurses' accommodation there as it at present exists, because of the object-lesson the Queen might thus acquire in what to avoid and what to aim at.

Perhaps those responsible may delay destroying the lamentable evidence of the want of artistic taste and practical knowledge to which we here call attention, that the fullest opportunity may be afforded for arrangements to be made for the Royal inspection on an early date. We have no doubt of the fruitful results which will accrue should it be found possible to bring it about. Doubtless the managers of the two lagging hospitals will prefer efficiency to delay, and so Glasgow nurses' homes may be brought at once equally up to standard.

